# Dysregulated autoantibodies targeting vaso- and immunoregulatory receptors in Post COVID Syndrome correlate with symptom severity

**DOI:** 10.3389/fimmu.2022.981532

**Published:** 2022-09-27

**Authors:** Franziska Sotzny, Igor Salerno Filgueiras, Claudia Kedor, Helma Freitag, Kirsten Wittke, Sandra Bauer, Nuno Sepúlveda, Dennyson Leandro Mathias da Fonseca, Gabriela Crispim Baiocchi, Alexandre H. C. Marques, Myungjin Kim, Tanja Lange, Desirée Rodrigues Plaça, Finn Luebber, Frieder M. Paulus, Roberta De Vito, Igor Jurisica, Kai Schulze-Forster, Friedemann Paul, Judith Bellmann-Strobl, Rebekka Rust, Uta Hoppmann, Yehuda Shoenfeld, Gabriela Riemekasten, Harald Heidecke, Otavio Cabral-Marques, Carmen Scheibenbogen

**Affiliations:** ^1^ Institute for Medical Immunology, Charité – Universitätsmedizin Berlin, corporate member of Freie Universität Berlin and Humboldt Universität zu Berlin, Berlin, Germany; ^2^ Department of Immunology, Institute of Biomedical Sciences, University of São Paulo, São Paulo, Brazil; ^3^ Faculty of Mathematics and Information Science, Warsaw University of Technology, Warsaw, Poland; ^4^ CEAUL – Centro de Estatística e Aplicações da Universidade de Lisboa, Lisbon, Portugal; ^5^ Interunit PostGraduate Program on Bioinformatics, Institute of Mathematics and Statistics (IME), University of Sao Paulo, Sao Paulo, Brazil; ^6^ Data Science Initiative, Brown University, Providence, RI, United States; ^7^ Department of Rheumatology and Clinical Immunology, University of Lübeck, Lübeck, Germany; ^8^ Department of Clinical and Toxicological Analyses, School of Pharmaceutical Sciences, University of São Paulo, São Paulo, Brazil; ^9^ Department of Psychiatry and Psychotherapy, University of Lübeck, Lübeck, Germany; ^10^ Department of Biostatistics and the Data Science Initiative, Brown University, Providence, RI, United States; ^11^ Osteoarthritis Research Program, Division of Orthopedic Surgery, Schroeder Arthritis Institute, University Health Network, Toronto, ON, Canada; ^12^ Data Science Discovery Centre for Chronic Diseases, Krembil Research Institute, University Health Network, Toronto, ON, Canada; ^13^ Department of Medical Biophysics, University of Toronto, Toronto, ON, Canada; ^14^ Department of Computer Science, University of Toronto, Toronto, ON, Canada; ^15^ Institute of Neuroimmunology, Slovak Academy of Sciences, Bratislava, Slovakia; ^16^ CellTrend GmbH, Luckenwalde, Germany; ^17^ Experimental and Clinical Research Center, a cooperation between the Max Delbrück Center for Molecular Medicine in the Helmholtz Association and Charité Universitätsmedizin Berlin, Berlin, Germany; ^18^ Charité – Universitätsmedizin Berlin, corporate member of Freie Universität Berlin and Humboldt-Universität zu Berlin, Experimental and Clinical Research Center, Berlin, Germany; ^19^ Max Delbrück Center for Molecular Medicine in the Helmholtz Association (MDC), Berlin, Germany; ^20^ NeuroCure Clinical Research Center, Charité – Universitätsmedizin Berlin, corporate member of Freie Universität Berlin and Humboldt-Universität zu Berlin, Berlin, Germany; ^21^ Zabludowicz Center for Autoimmune Diseases, Sheba Medical Center, Affiliated with the Sackler Faculty of Medicine, Tel-Aviv University, Tel-Hashomer, Israel; ^22^ Ariel University, Ariel, Israel; ^23^ Network of Immunity in Infection, Malignancy, and Autoimmunity (NIIMA), Universal Scientific Education and Research Network (USERN), Sao Paulo, Brazil; ^24^ Department of Pharmacy, Federal University of Rio Grande do Norte, Natal, RN, Brazil

**Keywords:** autoantibodies, COVID-19, post COVID syndrome, Chronic Fatigue Syndrome, ME/CFS,, G-protein coupled receptor, autonomic nervous system, renin-angiotensin system

## Abstract

Most patients with Post COVID Syndrome (PCS) present with a plethora of symptoms without clear evidence of organ dysfunction. A subset of them fulfills diagnostic criteria of myalgic encephalomyelitis/chronic fatigue syndrome (ME/CFS). Symptom severity of ME/CFS correlates with natural regulatory autoantibody (AAB) levels targeting several G-protein coupled receptors (GPCR). In this exploratory study, we analyzed serum AAB levels against vaso- and immunoregulatory receptors, mostly GPCRs, in 80 PCS patients following mild-to-moderate COVID-19, with 40 of them fulfilling diagnostic criteria of ME/CFS. Healthy seronegative (n=38) and asymptomatic post COVID-19 controls (n=40) were also included in the study as control groups. We found lower levels for various AABs in PCS compared to at least one control group, accompanied by alterations in the correlations among AABs. Classification using random forest indicated AABs targeting ADRB2, STAB1, and ADRA2A as the strongest classifiers (AABs stratifying patients according to disease outcomes) of post COVID-19 outcomes. Several AABs correlated with symptom severity in PCS groups. Remarkably, severity of fatigue and vasomotor symptoms were associated with ADRB2 AAB levels in PCS/ME/CFS patients. Our study identified dysregulation of AAB against various receptors involved in the autonomous nervous system (ANS), vaso-, and immunoregulation and their correlation with symptom severity, pointing to their role in the pathogenesis of PCS.

## Introduction

Post COVID syndrome (PCS) following mild-to-moderate coronavirus disease 2019 (COVID-19) with persistent symptoms for more than six months affecting everyday functioning is reported in 10-20% of patients (1–4). PCS symptoms are diverse, with debilitating fatigue, post-exertional malaise (PEM), difficulties in breathing, pain, and cognitive dysfunction as the most frequently reported (1). We found in our observational study that half of the PCS patients with moderate to severe fatigue and exertional intolerance fulfill the Canadian consensus criteria (CCC) for myalgic encephalomyelitis/chronic fatigue syndrome (ME/CFS) (5–7). These patients were referred to as PCS/ME/CFS and the others as PCS/non-ME/CFS (5). Mechanisms of PCS remain poorly understood, but some first evidence point to both immune and vascular dysregulation (8–13). Prothrombotic autoantibodies (AAB) against anti-phospholipid and anti-type I interferon were among the first to be described in acute COVID-19 patients (14–16). Wang *et al.* showed elevated functional AAB levels directed against extracellular antigens with a high prevalence of AAB against immunomodulatory proteins like cytokines, chemokines, and others (17). In addition, AABs against the vasoregulatory renin-angiotensin-system (RAS)-related proteins Angiotensin-converting enzyme 2 (ACE2) and angiotensin type-1 receptor (AGTR1) were increased in acute COVID-19 patients and associated with the disease severity (18). More recently, we showed elevated levels of AAB directed against several vaso- and immunoregulatory G-protein coupled receptors (GPCRs), including RAS-related proteins, in moderate and severe acute COVID-19 patients being associated with clinical severity of COVID-19 (19). First studies also found AABs in PCS patients. Antigen array chip analysis detected, among others, AABs against IL2, CD8B, Thyroglobulin, and interferons in PCS patients, also found in acute COVID-19 (20). In addition, AAB levels against the cyclic citrullinated peptide, associated with rheumatoid arthritis, against the anti-tissue transglutaminase, associated with celiac disease (21), and against the desmoglein-2, previously described in arrhythmogenic right ventricular cardiomyopathy (22) were reported in post COVID-19 patients. Moreover, in a first study, functional AABs against vasoregulatory GPCRs were detected in post COVID-19 patients (23).

AABs against GPCRs are part of normal human physiology. These AABs are dysregulated in various autoimmune and non-autoimmune diseases (24). They can induce or alter signaling and play an essential role in regulating autonomic nervous system (ANS) as well as endothelial and immune cell function, which could also be of relevance in COVID-19. For example, AABs directed to the AGTR1 induced skin and lung inflammation and were one of the best AABs discriminating mild from severe COVID-19 patients (19, 25). Therefore, GPCR AABs may be useful as biomarkers indicating activation or alteration of respective receptors and pathways (26). In ME/CFS, there is evidence for an altered GPCR AAB network with disease-specific AAB correlations (27–29). AAB levels against β1 and β2 adrenergic receptors (ADRB1/2), as well as muscarinic acetylcholine receptors M3 and M4 (CHRM3/4) measured by ELISA, were elevated in a subgroup of ME/CFS patients (27, 28). Moreover, AABs against ADRB2 and CHRM4 significantly declined in clinical responders but not in non-responders receiving rituximab treatment for B-cell depletion (28). Elevated levels of CHRM1 AABs were described in ME/CFS patients in association with muscle weakness and neurocognitive impairment (30). Further, AABs against several vasoregulatory GPCRs measured by ELISA were associated with key symptoms of fatigue and muscle pain in postinfectious ME/CFS patients (29). In conclusion, these studies indicate that AAB against the ADRs and CHRMs are associated with ME/CFS (28–30).

Here we investigate levels of immunoglobulin (Ig)G AAB directed against vaso- and immunoregulatory receptors, including members of the classical RAS (AGTR1/2, BDKRB1) as well as the counter-regulatory ACE2/MAS1 axis, against endothelin receptors (EDNRA/B), receptors related to the ANS (ADRs, CHRMs, CHRN), and the protease-activated receptor F2R/PAR-1, the chemokine receptor CXCR3 and the scavenger receptor stabilin-1 (STAB1). Thus, we aim to get insight into a potential dysregulation of the AAB and/or targets, most of them GPCRs, and the linked pathways in PCS. Further, we correlated the AAB levels with symptom severity. Importantly, we found an alteration of various AABs in PCS patients compared to post COVID-19 and seronegative healthy controls (PCHC and HC), as well as associations of AABs with clinical symptom severity.

## Methods

### Patients

Sera of 80 patients with PCS following mild-to-moderate COVID-19 and 78 healthy individuals were studied.

PCS patients were enrolled at the Charité Fatigue Center within an observational cohort study between August 2020 and July 2021. PCS patients had a confirmed diagnosis of mild to moderate COVID-19 (PCR or serology) and suffer from persistent moderate to severe fatigue and exertion intolerance post severe acute respiratory syndrome coronavirus type 2 (SARS-CoV-2) infection. Patients with relevant cardiac, respiratory, neurological, or psychiatric comorbidity, preexisting fatigue, or evidence of organ dysfunction were excluded (5). Patients were diagnosed at least six months following COVID-19. In the case of 17 of 80 patients, the diagnosis was retrospectively confirmed at maximum two months following blood sampling. Diagnosis of ME/CFS was based on the 2003 CCC, being the recommended diagnostic criteria for ME/CFS by EUROMENE for research purposes (6, 7). PCS/CFS patients fulfilling CCC suffer from persistent fatigue for at least six months, PEM, sleep disturbance, pain, at least two neurological or cognitive manifestations, and at least one symptom from two of the following categories: autonomic, neuroendocrine, or immune manifestations (7). In contrast to the original CCC classification and in accordance with the studies of Cotler et al., 2018, a minimum of 14 hours of PEM duration was required for diagnosing ME/CFS [(31) and **Table 1**]. Forty patients fulfilled the criteria for ME/CFS (6), referred to as PCS/ME/CFS, and the other patients as PCS/non-ME/CFS, which comprise the COVID-19 outcomes of interest to this manuscript. Patient groups were matched for disease duration at the time of blood sampling (**Table 1**).

**Table 1:** Characteristics of study groups.

Study group	PCS/ME/CFS(*n*=40)	PCS/non-ME/CFS(*n*=40)	PCHC(*n*=40)	HC(*n*=38)	*p-value*
Age, median (range) [years]	46.5 (24-62)	40 (22-67)	35 (21-66)	38 (19-64)	**0.0103** (**p**_PCS/CFSvs.PCHC_ =**0.0081**)
Female sex, *n*	33	28	23	27	0.1118
COVID-19 severity	moderate: 8 mild: 32	moderate: 8 mild: 32	NA	NA	>0,9999
Months after COVID infection, median (range)	7 (4-14)	7 (4-13)	5.5 (4-10)	NA	**0.0049** (**p**_PCS/CFSvs.PCHC_ =**0.0177;** **p**_PCS/non-CFSvs.PCHC_ =**0.0114**)
PEM [n]	40	38	NA	NA	0.1521
PEM >14h [n]	40	11	NA	NA	**<0.0001**
PEM score median (range)	34 (15-46)	24 (1-40)	NA	NA	**<0.0001**
Chalder Fatigue Scale median (range)	27 (18-33)	25 (14-32)	NA	NA	**0.0234**
Bell Disability Scale median (range)	40 (10-80)	50 (30-90)	NA	NA	**0.0017**
SF36 Physical Functioning median (range)	33 (6-65)	37.5 (10-72)	NA	NA	**0.0287**
Symptoms severity scores median (range)					
Fatigue	8 (3-10)	7.5 (2-10)	NA	NA	0.2538
Cognitive score	5 (2-10)	4.85 (1-7.3)	NA	NA	0.4073
Headache	6 (1-10)	5 (1-9)	NA	NA	0.2466
Muscle pain	6 (1-10)	6 (1-10)	NA	NA	0.1728
Immune score	3.3 (1-9.3)	2.15 (1-8)	NA	NA	**0.0071**
COMPASS-31 total, median (range)	36.05 (7-65.16)	29.05 (2.5-62.4)	NA	NA	0.3793
COMPASS-31 orthostatic, median (range)	24 (0-40)	20 (0-40)	NA	NA	0.3958
COMPASS-31 vasomotor, median (range)	0 (0-4.2)	0 (0-4.2)	NA	NA	0.2573
COMPASS-31 secretomotor, median (range)	6.4 (0-15)	2.1 (0-12.86)	NA	NA	0.0857
COMPASS-31 gastrointestinal, median (range)	5.8 (0-15.2)	6.2 (0-17)	NA	NA	0.4670
COMPASS-31 bladder, median (range)	0 (0-5.6)	0 (0-4.4)	NA	NA	0.2678
COMPASS-31 pupillomotor, median (range)	1.483 (0-3.7)	1.3 (0-3)	NA	NA	0.6486
IgG total, median (IQR) [g/l]	10.85 (8.9-14.28)	10.3 (9.45-13.13)	9.7 (8-11.28)	11 (8.65-14.23)	0.1518

Kruskal-Wallis test was used when comparing more than two groups and Mann-Whitney-U rank-sum-test when comparing two groups. If the Kruskal-Wallis test results in p<0.05, the post hoc Dunn’s test was performed, and p-values ≤0.5 were added to the table in brackets. Chi-square test was used to compare the distribution of gender, COVID-19 severity, and PEM. A two sided p-value ≤ 0.05 was considered statistically significant (in bold). IQR, interquartile range; NA, not assessed.

Healthy individuals include 40 PCHC, all after mild-to-moderate COVID-19, and 38 SARS-CoV-2-spike-IgG-negative HC without COVID-19 history. Serum SARS-CoV-2-spike-IgG was determined using Anti-SARS-CoV-2 ELISA (IgG) purchased from Euroimmun (Lübeck, Germany) according to manufacturers’ protocol. All PCS patients and healthy individuals selected had not received SARS-CoV-2 vaccination. Samples of PCHC were collected from July 2020 until June 2021 with a similar period following COVID-19 compared to PCS patients (**Table 1**). Since women are more susceptible to PCS and ME/CFS than men (6, 32) study groups were matched for gender but not for age because of the limited number of participants. The study was approved by the Ethics Committee of Charité Universitätsmedizin Berlin in accordance with the 1964 Declaration of Helsinki and its later amendments. All participants signed informed consent.

For comparative statistical analysis of patient characteristics, the Kruskal-Wallis test with *post hoc* Dunn’s test was performed using GraphPad Prism 6.0. The Chi-square test was used for comparative analysis of the distribution of gender, COVID-19 severity, and PEM of study groups. A two-sided p-value ≤ 0.05 was considered statistically significant.

### Determination of AAB

Whole blood samples from each subject were allowed to clot at room temperature and then centrifuged at 2000 x g for 15 min in a refrigerated centrifuge. The serum was purified and stored at −35°C. IgG AAB against Angiotensin II receptor type 1/2 (AGTR1/2), ACE2, MAS1, Bradykinin receptor B1 (BDKRB1), EDNRA/B, ADRA1/2A, ADRB1/2, CHRM1-5, nicotinic acetylcholine receptor subunit alpha 1 (CHRNA1), F2R/PAR-1, STAB1, and CXCR3 were measured using respective sandwich ELISA kits by CellTrend GmbH (Luckenwalde, Germany) as described before (19, 29). In brief, serum samples were diluted at a 1:100 ratio for ELISA. The AAB levels were calculated as arbitrary units (U) by extrapolating from the standard curve of five standards ranging from 2.5 to 40 U/ml. The ELISA kits were validated in accordance with the Food and Drug Administration’s Guidance for Industry: Bioanalytical Method Validation. The total serum IgG concentration was analyzed using Human IgG ELISABASIC Kit purchased from MABTECH AB (Nacka Strand, Sweden) according to the manufacturer’s protocol.

### Symptom assessment by questionnaires

The severity of fatigue and other key symptoms were measured using a Likert Scale (1 = no symptoms up to 10 = most severe symptoms) by the patients. The severity of fatigue was also evaluated using the Chalder Fatigue Scale from zero (no fatigue) to 33 (heavy fatigue) (33). PEM was assessed by a questionnaire (31), which describes an intolerance to mental and physical exertion triggering an aggravation of symptoms typically lasting for more than 14 hours up to several days (34). PEM score ranges from zero (no PEM) to 46 (frequent, severe, and long PEM). In addition, disability was assessed using the Bell score ranging from zero (total loss of self-dependence) to 100 (without restrictions) (35). Physical activities of daily life were assessed by Short Form Health Survey 36 (SF-36) ranging from zero (greatest possible health restrictions) to 100 (no health restrictions) (36). Quantification of the key symptoms ranges from one (no symptoms) to ten (extreme symptoms) (37). Symptoms of autonomic dysfunction were assessed by the Composite Autonomic Symptom Score 31 (COMPASS 31), ranging from zero (without symptoms) to 100 (strongest symptoms) (38).

The Mann-Whitney-U rank-sum-test was performed for comparative statistical analysis using GraphPad Prism 6.0. A two-sided p-value ≤ 0.05 was considered statistically significant.

### Visualization of autoantibody targets and pathways interactions

We searched for physical protein interactions (PPIs) between AAB targets using Integrated Interactions Database, IID version 2021-05 [http://ophid.utoronto.ca/iid (39)], combined with interactions from virus-human interactome (40). The interactions were then used to construct a network figure prepared using NAViGaTOR version 3.0.16 (41). Interactions between the autoantibody targets and their respective Gene Ontology (GO) biological processes (BP) were visualized by NAViGaTOR, as well as their interactions with human and SARS-CoV-2 molecules that are involved in the infection. Comprehensive pathway analysis of the 20 autoantibody targets and their interactors was performed using pathDIP version 4.1 [http://ophid.utoronto.ca/pathDIP (42)]. For the circular plot, emapplots, and enrichment, R version 4.0.5 (43), R studio Version 1.3.959 (44) were utilized, as well as Circos (45) and the R packages networkD3, ReactomePA, clusterProfiler, ggplot2, and viridis (46–51). After filtering the pathways for the most general level of the ontologies, up to 15 of the most significant pathways were plotted in the emapplots. Given the biological processes ontology, we chose ten pathways based on their relevance for the discussion out of the most significant ones for each target and performed a circular plot. The R package openxlsx was used throughout the analysis to read and write files (52).

### Pairwise comparison and differences in autoantibody concentrations

Differences in autoantibody levels were assessed by the Kruskal-Wallis test followed by Dunn test and adjusted for false discovery rate (FDR) *via* the R package rstatix (53). For each of the AABs, log2-transformed data was used for better visualization. Boxplots were generated using the R packages ggplot2, ggpubr, and lemon and plotted based on median and interquartile range (48, 54, 55). Adjusted p-values were represented by: *p<0.05; **p<0.01; ***p<0.001; ****p<0.0001.

### Regression analysis

AAB levels were modeled *via* the generalized additive models for location, scale, and shape (GAMLSS) (56). After analyzing the residual plots, Lognormal was chosen as the best fitting distribution for the data. To study potential confounders that may influence the outcomes and the autoantibody levels, i.e., age, sex (female taken as reference), and time since infection, which were considered covariables to model the AABs mean distribution in regards to clinical classification. HC group’s time since infection was considered zero, and five missing values for PCS/ME/CFS patients were substituted by the group’s median, seven.

### Principal component analysis

Principal Component Analysis (PCA) using a single value decomposition of the data matrix (57, 58) was used to measure the stratification of HC/PCHC and PCS based on the donors’ autoantibody levels. Prior to the analysis, the raw AAB levels were log2-transformed. PCA was performed using the R package factoextra and the prcomp function, in which data was centered and scaled (59). The number of principal components was chosen according to Kaiser Criterion (60).

### Ranking autoantibodies by Random Forest

We used the R package Random Forest (version 4.6.14) (61) to rank the AABs as classifiers of disease outcomes. We trained the random forest model using the 20 AAB levels (for which the number of variables randomly selected for each split, the mtry, was specified as 4), and five thousand trees were used for the classification. Follow-up analysis was conducted with the Gini decrease, number of nodes, and mean minimum depth as criteria to determine variable importance. Receiver Operating Characteristic (ROC) curve (and its area under the curve) and out-of-bags error rate were used to evaluate the stratification of disease groups as previously described (19).

### Autoantibody correlation signatures

Circular networks based on Spearman’s rank correlation coefficient were constructed with the R package qgraph (62), using the Log2-transformed AAB levels.

### Correlation analysis of AAB with clinical symptoms

Correlation analysis of AAB with clinical symptoms was performed using Spearman’s rank correlation using GraphPad Prism 6.0 (GraphPad Software, San Diego, CA). A two-sided p-value ≤ 0.05 was considered statistically significant.

## Results

### Study population

**Figure 1:**
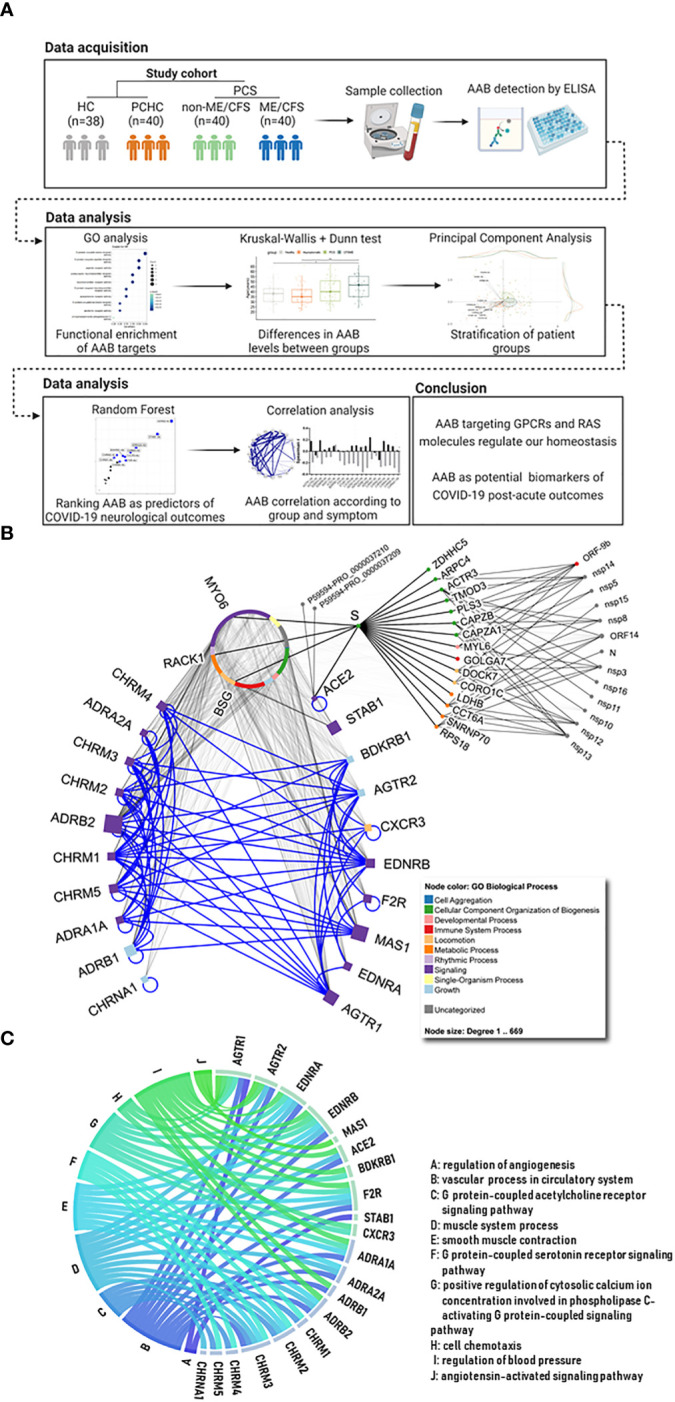
Study workflow and description of autoantibody targets. **(A)** After data acquisition, different statistical analyses (written on the top) were carried out in order to characterize the signature of autoantibodies (AAB) against G protein coupled receptors (GPCRs) and COVID-19-associated molecules (e.g. renin-angiotensin system (RAS)) in Post COVID Syndrome (PCS) when compared with healthy controls (HC) and post COVID-19 healthy controls (PCHC). Created with Biorender. **(B)** The 10 squares on the left represent autonomic nervous system (ANS) related receptors, while the 10 on the right show non-ANS molecules and receptors (e.g. RAS, immune and circulatory systems). Blue edges in the network highlight the interactions among the AAB targets, while gray edges represent other interactions. Node colors map to Gene Ontology (GO) biological processes (BPs) and node size corresponds to number of interacting partners for each target. Circular nodes represent human and SARS-CoV-2 molecules (as well as two Spike (S) proteins with unspecified roles) that are described in the IMEx coronavirus interactome. Circular organization of the proteins on the top middle of the image represent interacting partners of the AAB targets (names are omitted, except for 3 proteins that link ACE2 *via* S). **(C)** Circular plot with targets and relevant pathways they are associated to. Edge colors differ between each pathway. Edges representing AAB pathways are named from A to J, and the corresponding name is present in the list.

AABs of 80 PCS patients were measured. The majority of the PCS patients (64 of 80) had mild, and 16 had moderate COVID-19 (**Table 1**) due to pneumonia, according to WHO criteria (63). Forty patients fulfilled the CCC for ME/CFS (6, 7) referred to as PCS/ME/CFS, and the other patients as PCS/non-ME/CFS. Convalescent individuals who had COVID-19 (PCHC) during the same period and healthy seronegative individuals without a history of COVID-19 (HC) served as controls. The study design is shown in **Figure 1A**. **Table 1** summarizes the demographic characteristics of the study population. The study groups differ in age, with PCS/ME/CFS patients being on average 11.5 years older than PCHC. The median time interval since COVID-19 infection was seven months in patients and 5.5 months in PCHC, respectively. As required as diagnostic criteria for ME/CFS, all PCS/ME/CFS suffered from PEM with a minimum duration of 14 hours [(31) and **Table 1**]. 38 of 40 PCS/non-ME/CFS patients fulfilled the criteria for PEM, according to Cotler J. et al., 2018 (31) with 11 of them showing PEM for more than 14 hours. The PEM score measuring the severity and frequency of PEM was higher in PCS/ME/CFS than in PCS/non-ME/CFS patients. According to the Chalder Fatigue Scale, the level of fatigue was higher in PCS/ME/CFS patients than in PCS/non-ME/CFS ones. Patients’ disability assessed by the Bell Disability Scale and physical functioning assessed by SF36 was stronger impaired in PCS/ME/CFS patients compared to PCS/non-ME/CFS patients. However, the severity of fatigue, cognitive symptoms, headache, and muscle pain measured by symptom severity score did not significantly differ between PCS patient groups. Immune symptoms were more severe in PCS/ME/CFS than in PCS/non-ME/CFS. The median total COMPASS-31 score and the subdomains assessing autonomic function do not significantly differ between patient groups.

### Differences in the AAB levels between PCS/ME/CFS, PCS/non-ME/CFS and controls

The interactions of the AAB targets are represented in **Figure 1B**. The ten adrenergic and muscarinic receptors on the left side are related to the ANS and play a role in regulation of the vascular tone and circulation. The proteins plotted on the right side include members of the RAS System, RAS-related receptors, and further vaso- and immune-regulatory non-ANS proteins. Major biological processes in which these AAB targets are involved are shown in **Figure 1C**, and the most significant gene ontologies and their associations can be found in **Supplementary Figure 1**. The majority of the AAB targets are involved in the regulation of vessel tone, blood pressure, and muscle system processes.

**Figure 2:**
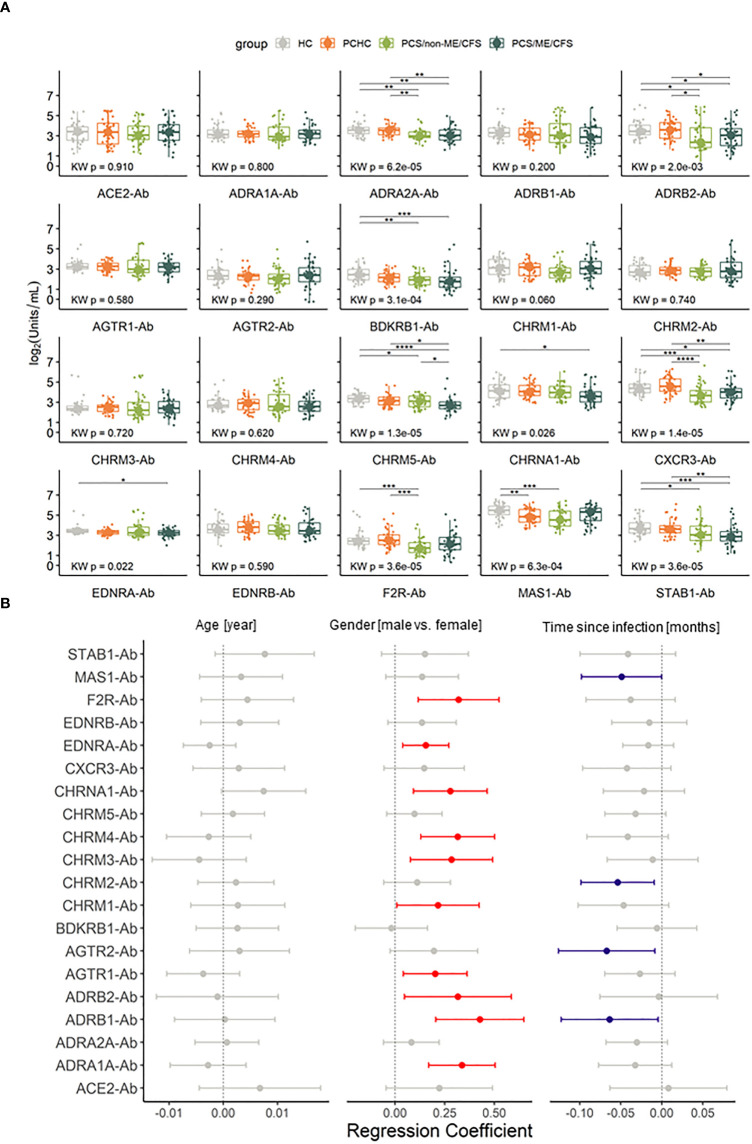
Autoantibodies (-Ab) against G protein coupled receptors (GPCR) and COVID-19-associated molecules are dysregulated during Post COVID Syndrome (PCS). **(A)** Box plots of Ab investigated in PCS patients with and without ME/CFS and healthy controls post or without COVID-19 history (PCHC or HC). Significance determined by Kruskal Wallis test followed by Dunn test as *post hoc*. Dunn test p values were corrected for FDR. Adjusted p-values are being represented by: *p.adj < 0.05; **p.adj <0.01; ***p.adj < 0.001; ****p.adj < 0.0001. Boxes represent the median and interquartile range (IQR). **(B)** Forest plot of regression coefficients for the confounding factors age in years, gender (reference being female) and time post COVID-19 in months considering 95% confidence interval (CI). Red dots and CI indicate that variable has a positive influence in the Ab level, blue dots and CI indicate a negative influence and gray ones contain 0 in the confidence interval, therefore are taken as non significant.

The AABs do not satisfy the criteria for lognormal distribution after log2 data transformation, and because of that, non-parametric tests were employed in this analysis. Significantly lower concentrations of ten AAB that target eight GPCRs, one ionotropic, and one scavenger receptor were found when comparing either PCS groups with healthy groups, namely: ADRA2A, ADRB2, BDKRB1, CHRM5, CHRNA1, CXCR3, EDNRA, F2R/PAR-1, MAS1 and STAB1 (**Figure 2A**; **Supplementary Table 1**).

Furthermore, we conducted regression analysis to evaluate the influence of age, gender, or time post-infection on AAB levels. Considering these factors, age did not significantly affect the AAB levels. In turn, while we observed a general tendency for higher AAB levels in males, there is a trend for AAB levels to decrease with time post-infection. These confounding effectors significantly affected the levels of 13 specific AAB, with 9 AAB levels being affected by gender, 3 AAB levels by disease duration, and ADRB1 antibody (-Ab) levels by both (**Figure 2B** and **Supplementary Table 2**). Most significant regression coefficients were positive by adjusting for age, gender, and time post-infection and in regards to PCS/ME/CFS, suggesting that lower AAB levels are associated with this phenotype. In contrast, for F2R/PAR-1- and CHRM1-Ab, the regression coefficient was negative in PCS/non-ME/CFS, indicating that lower levels of these AABs were associated with the non-ME/CFS phenotype in regards to PCS/ME/CFS, as well as for CHRM2 in HC (**Supplementary Table 3**).

Compared to both HC and PCHC, AAB concentrations against ADRA2A, ADRB2, STAB1, and CXCR3 significantly decrease in both PCS/non-ME/CFS and PCS/ME/CFS (**Figure 2A**). Diminished levels of CHRM5-Ab and CHRNA1-Ab were found in PCS/ME/CFS while reduced F2R/PAR-1 AAB in PCS/non-ME/CFS exclusively. After adjustment for the potential confounders, sex, age, and time since infection, we found in addition to CHRM5-Ab significant differences with higher levels of AABs against CHRM1 and F2R/PAR-1 while lower levels of ADRB1, CHRNA1, and EDNRA in PCS/ME/CFS than in PCS/non-ME/CFS groups (**Figure 2A** and **Supplementary Table 3**). Therefore, indicating that PCS patients with and without ME/CFS only barely differ in their AAB levels, at least when considering the concentrations of the 20 AAB. Furthermore, only for MAS1-Ab, we observed different levels between PCHC and HC (**Figure 2A**). These results suggest a similar profile among healthy and asymptomatic post COVID-19 donors. There were no significant differences in levels of total IgG among patients and controls (**Table 1**). Taken together, we found distinct AAB profiles for each of the studied conditions.

### Stratification of study groups based on AAB data

**Figure 3:**
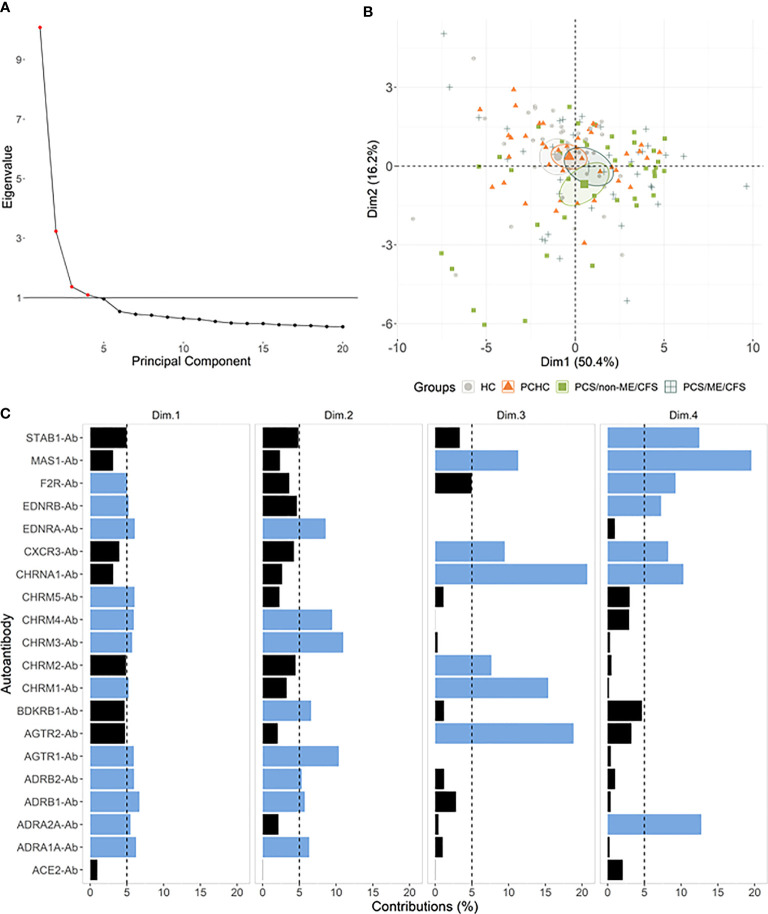
Autoantibodies (-Ab) stratify patients by post-acute COVID-19 outcomes. **(A)** Principal component analysis (PCA) with spectral decomposition based on logarithmic values of 20 Abs show the stratification of the four studied groups. Variables pointing to the same sense of the corresponding principal components are positive correlated. Small ellipses are the concentration around the mean points of each group. **(B)** Graphs of variables (Abs) obtained by PCA of all individuals in this study. **(C)** Barplot with the contribution percentages of each variable to each dimension. A black dashed line is plotted on the 5% mark, and blue bars indicate a contribution higher than 5%.

Next, we performed principal component analysis (PCA) to examine the association between AABs (variables) and individuals (observations) while stratifying groups based on the AAB level. According to the Kaiser criterion, the first four principal components were considered for the analysis (**Figure 3A**). For dimensions 1 and 2, there was some overlap between the groups, except for HC+PCS/non-ME/CFS and PCHC+PCS/non-ME/CFS (**Figure 3B**). The contribution of each AAB across the PCA dimensions is shown in **Figure 3C**. Noteworthy, no AABs are negatively related to the first dimension (**Supplementary Figure 2A**). PCA plots using different combinations based on dimensions 1 to 4 revealed a similar AAB profile between control groups HC and PCHC as well as between PCS groups (**Figure 2B**; **Supplementary Figures 2B–F**).

**Figure 4:**
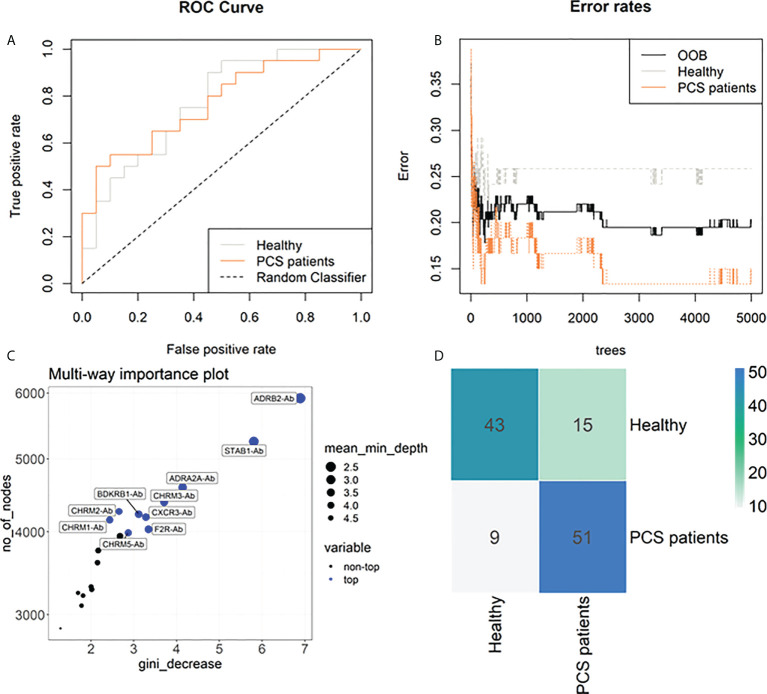
Machine learning classification of study groups based on autoantibodies. **(A)** Receiver operating characteristic (ROC) curves of 20 antibodies (Abs) with an area under the curve (AUC) of 77% for healthy individuals and 77% for PCS patient group. **(B)** Stable curve showing number of trees and out-of-bag (OOB) error rate of 20.34%. **(C)** Variable importance score plot based on Gini decrease and number (no) of nodes, and the mean of minimum depth for each Ab, showing which variable presents a higher score in classifying COVID-19 post-acute infection outcomes. **(D)** Heatmap of the confusion matrix. Numbers represent the amount of occurrences that happened when training the random forest model in predicted (row) vs actual classification (column), therefore the blueish diagonal identifies the hits, while other cells are mismatches.

To further investigate the potential of AAB to classify PCS patients, we carried out a Random Forest analysis, joining HC and PCHC groups (named as Healthy) as well as both PCS groups (referred to as PCS patients) due to their similar AAB pattern. This approach indicated an out-of-bag (OOB) error rate of 20.34% (25.86% for Healthy and 15% for PCS patients) and an area under the curve (AUC) of 0.77 for each group (**Figures 4A, B**). In addition, the Random Forest model ranked the AABs based on their ability to discriminate between study groups, identifying ADRB2-Ab, STAB1-Ab, and ADRA2A-Ab as the three most important classifiers (**Figure 4C**). In agreement with the PCA results, AABs were able to partially correctly classify the individuals into the Healthy and PCS groups (**Figure 4D**).

### AAB correlation signature in study groups

**Figure 5:**
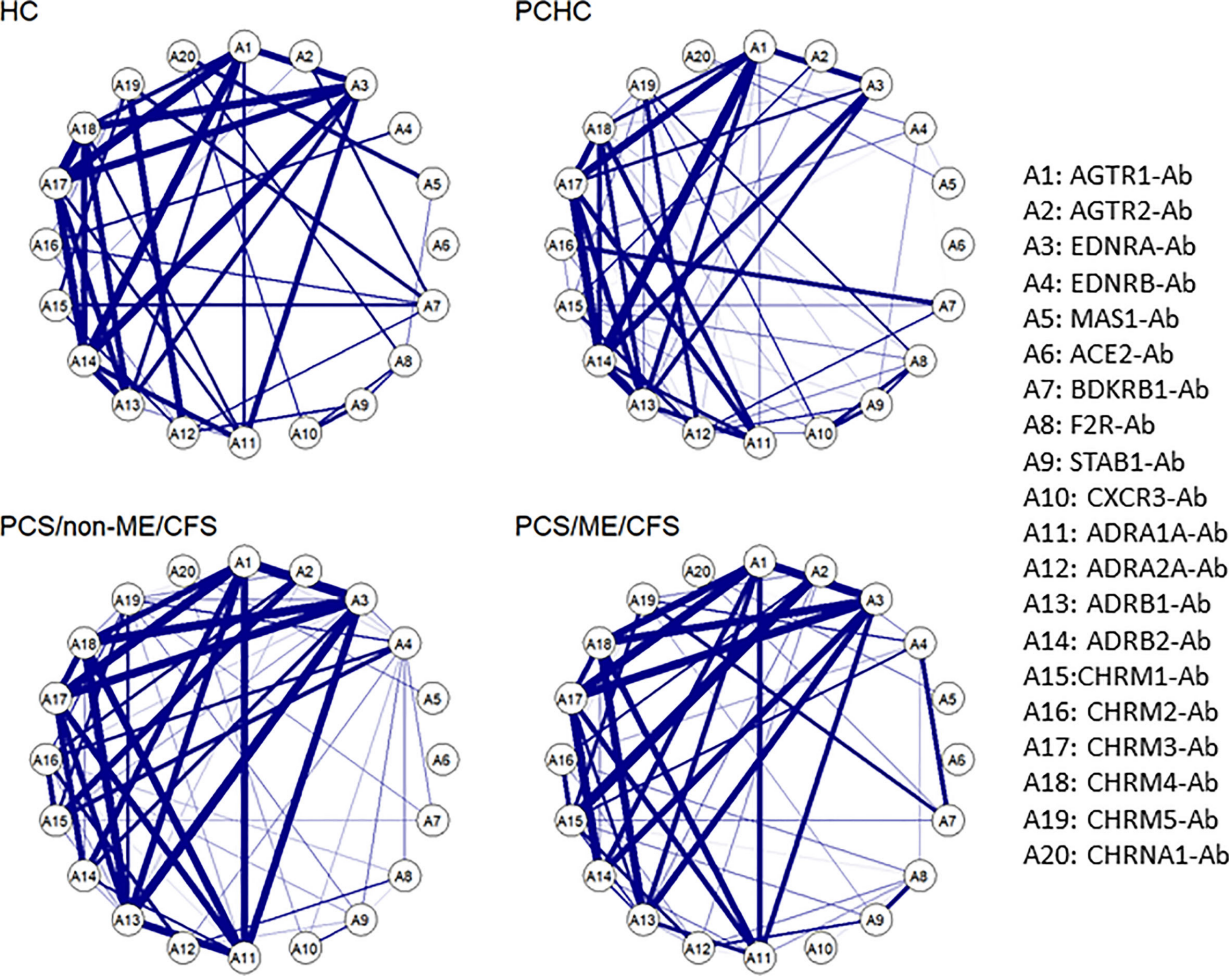
Autoantibody correlation signatures associate with post-acute infection outcome. Circular networks based on Spearman’s rank correlation for the level of the 20 autoantibodies (-Ab) in post COVID syndrome (PCS) patients with and without ME/CFS and healthy controls post or without COVID-19 history (PCHC or HC). There is a list with the abbreviations and the Abs names by the right side of the plot. Correlations greater than 0.6 are represented by the blue edges, and thicker edges imply greater correlations.

In our previous study, we reported that by clustering AAB correlation, it is possible to associate their signatures with immune homeostasis, leading to either a physiological or a pathological outcome (24). In this sense, our next step was to investigate how the data we analyzed behave regarding the AAB levels in each of the four groups. It is possible to identify a pattern characterizing every group, showing a strong correlation among AAB to ANS receptors. However, there are minor differences between the groups, namely the strong correlation of CHRM1-Ab with AGTR2-Ab and CHRM2-Ab in patients and between EDNBR-Ab and BDKRB1-Ab mainly in PCS/ME/CFS. Oppositely, a weakening in the correlation of ADRB2-Ab with ADRB1-Ab and CHRM4-Ab in patients in comparison with HC and PCHC was found. In addition, CHRM5-Ab correlated with ADRA2A-Ab in HC and PCHC only (**Figure 5**).

Although our regression analysis suggested an influence of gender and the disease duration as a confounding effect (**Figure 2B**), the low number of male patients per group in the cohort (see **Table 1**) precludes a robust correlation analysis in comparison to the female group.

### Correlation of AAB levels with clinical symptom scores

**Figure 6:**
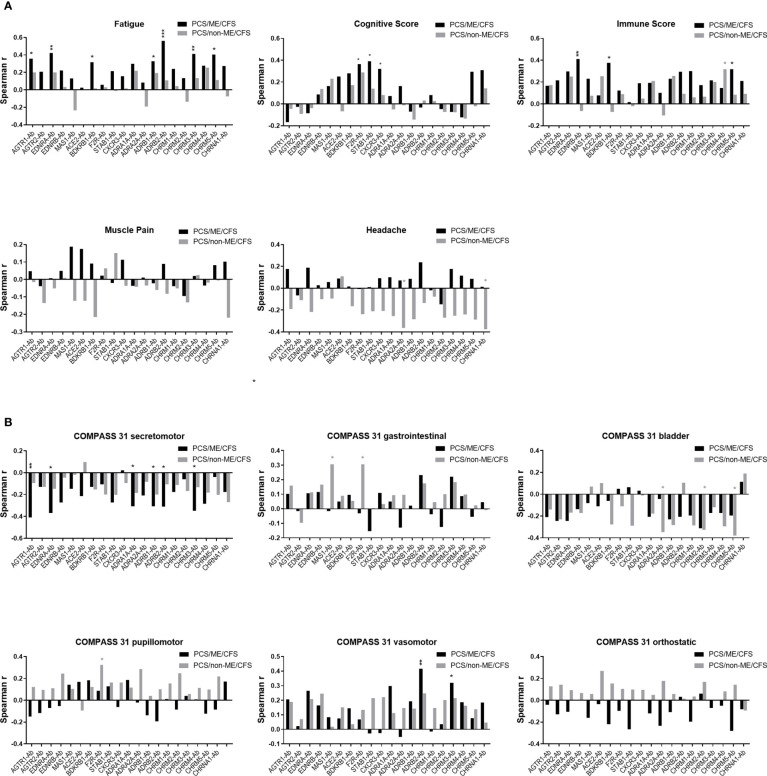
Correlation between autoantibody (-Ab) levels and clinical scores. Plots represent Spearman correlation coefficient (r) of correlation of Abs with **(A)** symptom scores and **(B)** autonomic symptom score assessed by COMPASS-31 questionnaire of PCS/non-ME/CFS (grey) and PCS/ME/CFS (black) patients. p values represented by: *p < 0.05, **p< 0.01 and ***p<0.001.

Correlation coefficients of symptom severity with AAB levels in PCS/ME/CFS and PCS patients are shown in **Figure 6A**. In PCS/ME/CFS, patients’ severity of fatigue correlated positively with levels of several AABs, including those against AGTR1, EDNRA, BDKRB1, ADRB1/2, CHRM3/5 (black bars). In contrast, the severity of cognitive symptoms correlated positively with F2R/PAR-1-, CXCR3-, and STAB1-Ab, and immune symptoms correlated with EDNRB-, BDKRB1-, and CHRM5-Ab in PCS/ME/CFS, while the severity of muscle pain and headache showed no significant correlations. None of these correlations were significant in PCS/non-ME/CFS (grey bars). In this cohort, there were only correlations of CHRM4-Ab with immune symptoms and of ADRB1- and CHRNA1-Ab correlated with headache.

An association was also observed between AABs and the severity of autonomic symptoms assessed by the COMPASS 31 questionnaire (**Figure 6B**). In PCS/ME/CFS patients, the secretomotor symptoms (dry eyes, dry mouth) correlated negatively with levels of AABs against AGTR1, EDNRA, ADRA1A, ADRB1/2, and CHRM3 (black bars). Interestingly, a correlation was found between ADRB2-Ab and CHRM3-Ab with the vasomotor function (Raynaud symptoms) in PCS/ME/CFS patients. Likewise, none of these correlations were significant in PCS/non-ME/CFS (grey bars). In this cohort, we found correlations between gastrointestinal symptoms with MAS1-Ab and both gastrointestinal and pupillomotor symptoms with F2R/PAR-1-Ab and a negative correlation of bladder symptoms with ADRA2A and CHRM2/5-Ab.

## Discussion

Here we found in PCS patients altered levels of AABs directed against various receptors, mainly GPCRs, regulating ANS, as well as vascular and immune processes (**Figure 1C**). In contrast to our previous studies in acute COVID-19 with upregulation of several AABs (17, 19, 64), a profound downregulation of various AABs was detected, accompanied by alterations in the correlations among AABs. Furthermore, while the PCA results of our study revealed a partial stratification overlap between HC and PCHC, as well as both PCS groups, machine learning classification indicated AABs against ANS-related receptors ADRA2A and ADRB2 and the scavenger receptor STAB1 as the most important classifiers of PCS. Finally, we found strong correlations among the AABs and several associations of AABs with key symptoms of PCS.

By acting as ligands to their target receptors, AABs against GPCRs can modulate receptor signaling. In most functional studies, GPCR AABs binding to their corresponding receptors results in agonist stimulation (25, 65–70). Supporting this assumption, the first study in PCS patients showed agonistic effects of GPCR AABs, with ADRA1-, AGTR1- and ADRB2-Ab stimulating and EDNRA- and MAS1-Ab inhibiting the beating rate of cardiomyocytes of neonatal rats *in vitro* (23). AABs against GPCRs appeared to be dysregulated in many diseases and associated with clinical symptoms (24, 26, 29). Dysregulation of GPCR AABs may either indicate an altered function of AABs resulting in altered target receptor signaling and/or expression or indicate a homeostatic response to an upregulation or downregulation of the respective receptors and pathways (26).

Our study found significantly lower concentrations of ten out of 20 analyzed circulating AAB in PCS groups compared to healthy control groups. AABs reduced in PCS/ME/CFS or PCS/non-ME/CFS patients included AABs regulating vascular tone (ADRA2A, ADRB2, BDKRB1, MAS1, CHRM5, CHRNA1, EDNRA, F2R/PAR-1), STAB1 playing a role as scavenger receptor and regulating angiogenesis as well as the inflammatory chemokine receptor CXCR3. Except for CHRM5-Ab, differences in AAB levels between patient groups were found only after adjustment for age, gender, and disease duration (time after infection), indicating that PCS patients with and without ME/CFS only barely differ in their AAB levels.

Mechanisms of PCS remain poorly understood; however, some evidence points to both immune and vascular dysregulation (8–10). Both ongoing low-grade inflammation and impaired circulation and oxygen supply could explain many symptoms of PCS, including fatigue, cognitive impairment, dyspnea, or muscle pain upon exertion. Further marked autonomic dysfunction has been found in PCS (5, 71). We found AABs diminished in PCS patients, target receptors and pathways playing an essential role in ANS and/or vascular regulation and/or inflammation. Among them, AABs against ADRB2 are considered to play a crucial role in endothelial dysfunction in ME/CFS, as reviewed in Wirth 2020 (72). Catecholamines binding to ANS receptors ADRA1, ADRA2, and ADRB1 on vascular smooth muscle cells cause vasoconstriction, while ADRB2 mediates vasodilation. Thus, the downregulation of both ADRA2A-Ab and ADRB2-Ab observed in our study point to a dysregulation in the coordination of vasoregulation, which is in accordance with our random forest results. In this context, we found an agonistic effect of ADRB2-Ab in HC in a previous study, which was diminished in ME/CFS (70). Further, we found a sequence in Epstein–Barr virus (EBV) with high homology with ADRA2, which may induce crossreactive IgG (73). This finding may be of relevance in PCS, too, as EBV reactivation during COVID was identified as a risk factor for PCS (74). CHRM5-specific AABs were diminished in PCS/ME/CFS patients compared to healthy individuals and PCS/non-ME/CFS patients. Interestingly, CHRM5 seems to be an important regulator of cerebral blood flow (CBF) (75, 76). In ME/CFS, CBF was impaired at least in a subset of patients (77, 78) and negatively correlated with fatigue severity (79). The scavenger receptor stabilin plays an important role in maintaining vascular integrity by clearing infected apoptotic endothelial cells (80). Further, EDNRA, CHRNA, and F2R/PAR-1 play a role in vasoconstriction and RAS-related receptors BDKRB1 and MAS1 in vasodilation (81–83). Moreover, F2R/PAR-1, ADRB2, and CHRN play a role in inflammation, with F2R/PAR-1 exerting pro- and CHRN as well as ADRB2 anti-inflammatory responses (84–86). Therefore, our study strongly supports a vascular dysregulation in PCS.

Upregulation of CXCL10, the ligand of CXCR3, is associated with COVID-19 severity promoting chemoattraction *via* CXCR3 for activated lymphocytes and monocytes (87). The CXCL10-CXCR3 axis is also likely to play an essential role in COVID-19-induced tissue injury and fibrosis, including pulmonary and cardiac fibrosis, endothelitis, and endothelial damage. In Sjogren’s Syndrome, an autoimmune disease with a high prevalence of fatigue, anti-CXCR3 AAB levels were also diminished and negatively correlated with circulating lymphocyte counts (88).

The most intriguing finding in our study is the correlations between the levels of several AABs with the severity of fatigue, and cognitive and immune symptoms in PCS/ME/CFS patients, thus further pointing to a role of these AABs or their associated pathways in disease pathomechanism. The severity of fatigue correlated positively with levels of circulating AAB against vasoregulatory AGTR1, EDNRA, ADRB1/2, BDKRB1, and CHRM5, all downregulated in our study. This finding is similar to previous results in postinfectious non-COVID-19 ME/CFS in which severity of fatigue correlated with AABs against ADRB1/2, EDNRA, and AGTR1 (29); BDKRB1 and CHRM5 were not analyzed in this previous study. These correlations suggest that vascular dysregulation plays a role in fatigue in both ME/CFS cohorts. In line with this suggestion, we observed a strong correlation of levels of ADRB2 AAB with Raynaud symptoms in the PCS/ME/CFS cohort in the present study. Similarly, the negative correlation of the secretomotor symptoms (dry eyes, dry mouth) with levels of AAB against vasoregulatory receptors AGTR1, EDNRA, ADRA1A, ADRB1/2, and CHRM3 indicate a vascular mechanism. The severity of cognitive symptoms correlated with AABs against F2R/PAR-1, CXCR3, and STAB1 in PCS/ME/CFS in contrast to our previous results in postinfectious non-COVID-19 ME/CFS in which cognitive impairment correlated with EDNRA and AGTR1 mediating vasoconstriction (CXCR3 and Stabilin-1 were not analyzed). As the AAB targets F2R/PAR-1, CXCR3, and STAB1 are involved in inflammatory processes (89, 90) this finding points to a distinct inflammatory mechanism in cognitive impairment in PCS/ME/CFS in contrast to vasoconstriction in the previous cohort of non-COVID ME/CFS. As ME/CFS patients in our previous study had a median time since disease onset of 3 years, one possible explanation for this difference may be that early in the disease course, an inflammatory mechanism, while later a vasoregulatory mechanism is more relevant for cognitive impairment. None of these correlations observed in the PCS/ME/CFS cohort were found in patients with PCS/non-ME/CFS. Taken together, the levels of several AABs were positively associated with key symptoms of ME/CFS in the PCS/ME/CFS cohort. Whether AAB-target interactions have a functional effect that promotes disease symptoms or are a response to pathophysiological changes in these receptors and pathways remains to be elucidated in future studies.

The levels of several AAB were unexpectedly lower in PCS patients compared to control groups. In one previous study, especially functional active GPCR AABs were detected in post COVID patients and symptom-free individuals using a bioassay (23). In the present research, AABs binding to the specific receptors were measured using ELISA independently by their functional properties, and these findings may explain the differences in results. In previous reports on acute COVID-19 elevated levels of AABs were observed in patients suffering from moderate to severe but not mild disease compared to controls (18, 19). In contrast, most patients in the present study had mild COVID. We do not think that differences in the healthy control groups play a role as we did not observe different AAB levels, except for MAS1-Ab, in healthy controls with or without COVID-19 history. A difference in gender distribution may be a confounder, as we found that the male gender was associated with higher AAB levels in our cohort. Our finding of lower AABs in PCS patients also is in contrast to previous studies in ME/CFS (27, 28, 30). While PCS patients analyzed here were median seven months post-infection, ME/CFS patients in these previous studies were mainly analyzed much later in the disease course. Lower GPCR AAB levels were found in vascular diseases such as acute coronary syndrome or vasculitis and in progressive lung involvement in rheumatic disease, too (26, 88, 91). One explanation could be that lower levels of circulating AABs result from AAB binding to their target molecules upregulated in the post-infection inflammatory endothelium or tissue in PCS patients. Upon resolution of the inflammation, serum AAB levels may increase again. Our first results of analyses of these AABs in PCS patients at a later time point support this hypothesis. Sequential studies, including samples later during disease progression, are thus crucial to understand the kinetics of these AABs during the disease course. Another explanation for lower serum AAB levels might be anti-idiotype antibodies (anti-IDs) directed against the GPCR AABs. Enhanced GPCR AAB levels during acute COVID-19 may induce enhanced anti-IDs (19). A pathophysiological role of anti-IDs was shown in various autoimmune diseases, like myasthenia gravis and diabetes mellitus, and discussed for PCS (92, 93). Consequently, the anti-IDs-Ab –interaction may interfere with the binding of the AABs to their target receptors resulting in reduced serum levels measured by ELISA.

Interestingly AABs to the vaso- and immunoregulatory receptors CXCR3, CHRM5, BDKRB1, MAS1, AGTR1, F2R/PAR-1, and STAB1 were the most significant classifiers of acute COVID-19 severity in our recent study (19). Our findings suggest that dysregulation of these AABs and/or related pathways during acute COVID-19 may also play a role in PCS. The separation of the patient from healthy cohorts by PCA and random forest indicates that it may also be possible to use the AAB signature as a biomarker for PCS. However, this needs to be confirmed in further cohorts. Reduced levels of AAB, which were accompanied by a progressive disruption in their (statistical) relationships in PCS compared to HC/PCHC, are in accordance with recent works showing that AAB correlation signatures are associated with both normal physiological and pathological immune homeostasis (19, 24). The dysregulation of any biological process, such as the imbalance (reduction or elevation) of cytokines/chemokines, can affect the body’s equilibrium and homeostasis. Our data support the analogous concept, where an imbalance of the homeostasis of AAB relationships is probably an underlying pathological mechanism. Thus, the present work reinforces the idea that AAB targeting GPCRs are natural components of the human physiology that become dysregulated during inflammatory and autoimmune diseases. This work expands the comprehension of AAB biology by considering the importance of vaso- and immunoregulatory GPCR in human inflammatory and autoimmune diseases. It may open novel avenues for understanding new mechanisms of body homeostasis. In this context, mechanistic studies characterizing the functions of anti-GPCR AAB in patients with PCS are essential and may provide new therapeutic targets. The potential of therapies to deplete AABs with altered binding and function should be explored.

Limitations of our study are suggested by our regression analysis of the influence of gender and the disease duration as a confounding effect that needs to be considered and investigated in more detail. Further studies with larger cohorts and sequential samples are required before any conclusions on generalizability, and potential diagnostic suitability can be made. The lack of data on the functional properties of the AABs is a further limitation of our study.

## Data availability statement

The raw data supporting the conclusions of this article will be made available by the authors, without undue reservation.

## Ethics statement

The studies involving human participants were reviewed and approved by Charité ethical committee (EA2_067_20 for assessment of biomarkers, EA2_066_20 for the PA-COVID study). The patients/participants provided their written informed consent to participate in this study.

## Author contributions

CS and OC-M designed the study. CS, CK, KW, FP, JB-S, RR and UH diagnosed and enrolled the patients. SB performed the sample preparation. HH and KS-F determined the serum autoantibody concentrations. FS and HF managed the collection and maintenance of the clinical and laboratory data. FS, IF, HF, NS, AM, RV, IJ, TL, YS, GR, OC-M and CS provided scientific insights. FS, IF, DMdF, GB, MK, OC-M, NS performed the data analysis. FS, IF, HF, NS, DMdF, GB, DP, TL, FL, FMP, FP, GR, OC-M and CS interpreted and discussed the results. FS, IF, OC-M and CS wrote the manuscript. All authors have read, revised, and approved the final version of the manuscript.

## Funding

CS, FS, HF, CK and KW thank the Weidenhammer Zoebele and CS the Lost Voices Foundation, Germany for financial support. We also thank the São Paulo Research Foundation (FAPESP grants 2020/07972-1 to GB; 2018/18886-9, 2020/01688-0, and 2020/07069-0 to OC-M; 2020/16246-2 to DF; 2020/11710-2 to DP), and the Coordination for the Improvement of Higher Education Personnel (CAPES) Financial Code 001 (grant to IF) for financial support. IJ was supported in part by the grants from Ontario Research Fund (#34876), Natural Sciences Research Council (NSERC #203475) and Canada Foundation for Innovation (CFI #29272, #225404, #33536). NS received funding from FCT - Fundação para a Ciência e Tecnologia, Portugal (ref. grant: UIDB/00006/2020) and NAWA - Polish National Agency for Academic Exchange (ref. grant: PPN/ULM/2020/1/00069/U/00001).

## Acknowledgments

We are grateful to the patients who participated in this study despite their individual disease-related impairment.

## Conflict of interest

The authors declare that HH and KS-F are managing directors of CellTrend. CellTrend holds together with Charité a patent for the diagnostic use of AABs against ADRB2. CS has a consulting agreement with CellTrend. FP reports grants from the Guthy Jackson Charitable Foundation, during the conduct of the study.

The remaining authors declare that the research was conducted in the absence of any commercial or financial relationships that could be construed as a potential conflict of interest.

The handling editor MD declared a past co-authorship with the author YS.

## Publisher’s note

All claims expressed in this article are solely those of the authors and do not necessarily represent those of their affiliated organizations, or those of the publisher, the editors and the reviewers. Any product that may be evaluated in this article, or claim that may be made by its manufacturer, is not guaranteed or endorsed by the publisher.

## Abbreviations

AAB, autoantibody; ACE2, Angiotensin-Converting Enzyme 2; ADRA, adrenergic receptor alpha; ADRB, adrenergic receptor beta; AGTR, Angiotensin II receptor; ANS, Autonomic nervous system; Ang, Angiotensin; BDKRB1, Bradykinin receptor B1; CCC, Canadian consensus criteria; CHRM, muscarinic acetylcholine receptor; CHRN, nicotinic acetylcholine receptor; CXCR3, CXC chemokine receptor 3; ED, endotelial dysfunction; EDNRA/B, endothelin receptor type A/B; F2R/PAR-1, coagulation Factor II Thrombin receptor/Proteinase-activated receptor 1; GPCR, G-protein coupled receptor; HC, healthy control; IQR, interquartile range; Mas1, MAS receptor; ME/CFS, Myalgic Encephalomyelitis/Chronic Fatigue Syndrome; NA, not assessed; ND, not determined; PCHC, post COVID-19 healthy control; PCS, post COVID Syndrome; PEM, post-exertional malaise; STAB1, Stabilin-1.
