# Supplementary material for: Dysregulated autoantibodies targeting vaso- and immunoregulatory receptors in Post COVID Syndrome correlate with symptom severity
Source: Front Immunol. 2022 Sep 27;13:981532. doi: 10.3389/fimmu.2022.981532 (PMC9552223; doi:10.3389/fimmu.2022.981532)
Supplement: Supplementary file 1 [file DataSheet_1.pdf]

Supplementary Material

Autoantibodies targeting G-protein coupled receptor- and renin-angiotensin-system- molecules are dysregulated in Post COVID Syndrome and correlate with symptom severity

Franziska Sotzny[1*], Igor Salerno Filgueiras[2*], Claudia Kedor[1], Helma Freitag[1], Kirsten Wittke[1], Sandra Bauer[1], Nuno Sepúlveda[3,4], Dennyson Leandro Mathias da Fonseca[5], Gabriela Crispim Baiocchi[2], Alexandre HC Marques[2], Myungjin Kim[6], Tanja Lange[7], Desirée Rodrigues Plaça[8], Finn Luebber[7,9], Frieder M Paulus[9], Roberta De Vito[10], Igor Jurisica[11-15], Kai Schulze-Forster[16], Friedemann Paul[17-20], Judith Bellmann-Strobl[1,17-19], Rebekka Rust[1,16-18], Uta Hoppmann[1,16-18], Yehuda Shoenfeld[21,22], Gabriela Riemekasten[7], Harald Heidecke[16], Otavio Cabral-Marques [2,5,8,23,24*], Carmen Scheibenbogen[1*]

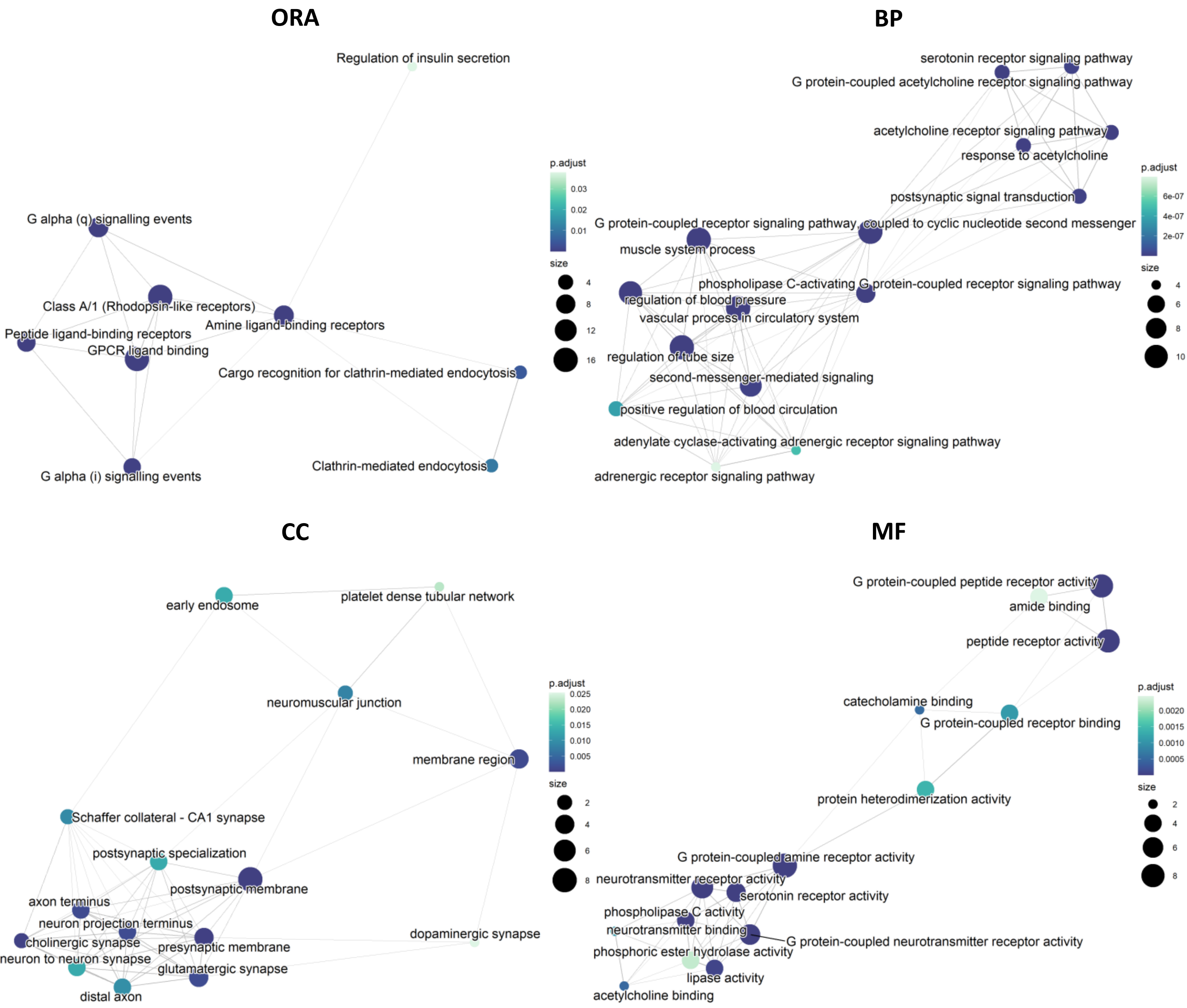

Supplementary Figure 1: Enrichment of autoantibodies targets and their functional similarities. Emapplots of biological functions shared by the targets of the 20 autoantibodies. Over Represented Analysis (ORA), and three categories of gene ontology: biological process (BP), cellular component (CC) and molecular function (MF); are displayed. Nodes represent a given pathway while their colors and sizes, the statistical significance and the amount of targets in the pathway, respectively. Edges are present when there is an association between the nodes. Only the 15 most significant pathways are shown.

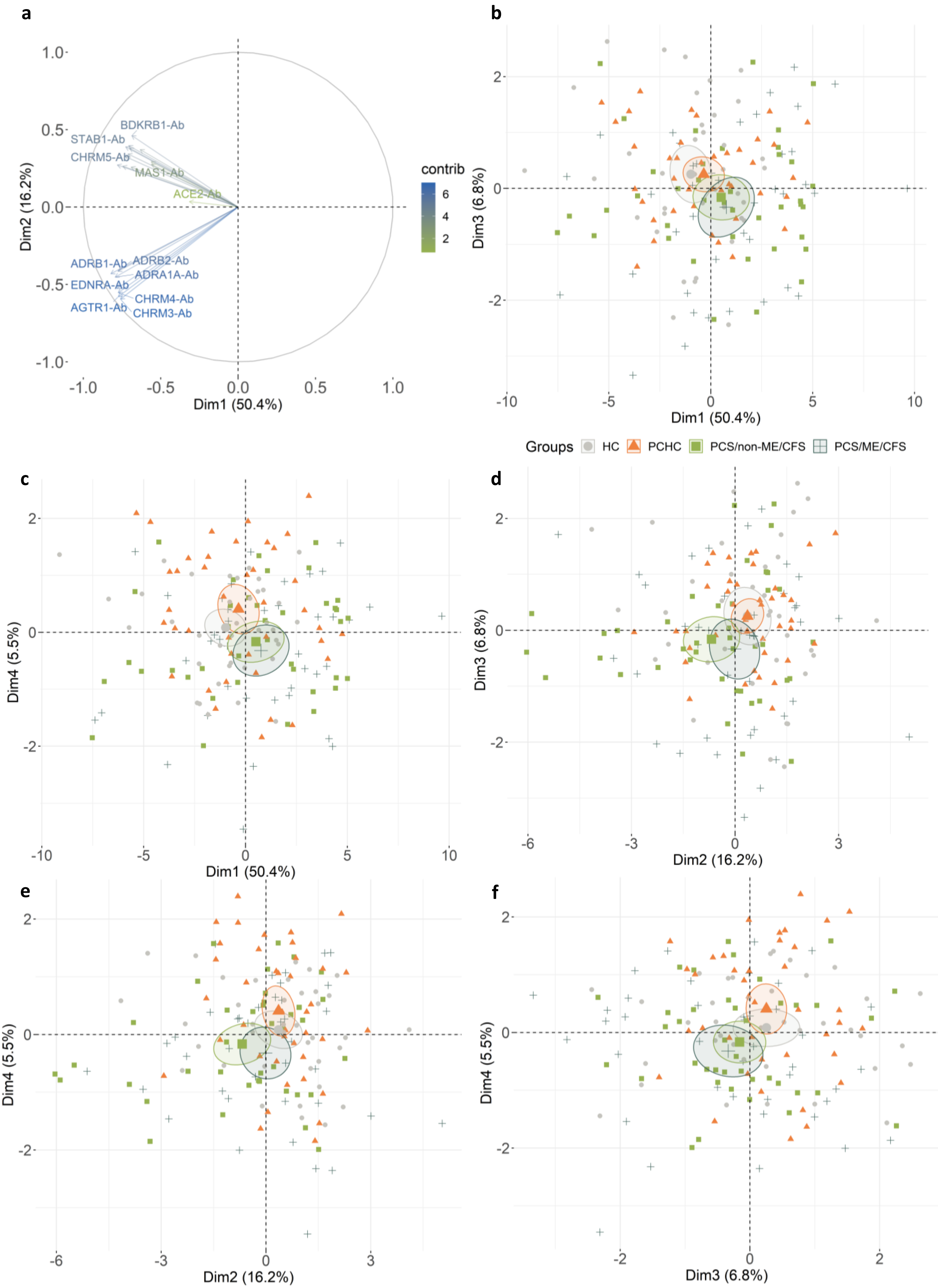

Supplementary Figure 2: PCA. (a) Variables' contribution to PC1 and PC2. **(b-f)** Plots of the combinations of the first four dimensions.

Supplementary table 1

aab	group1	group2	statistic	p	p.adj	p.adj.signif
ACE2-Ab	HC	PCHC	0,3424	0,7321	0,8964	ns
ACE2-Ab	HC	PCS/non-ME/CFS	-0,1557	0,8763	0,9419	ns
ACE2-Ab	HC	PCS/ME/CFS	0,4702	0,6382	0,8509	ns
ACE2-Ab	PCHC	PCS/non-ME/CFS	-0,5046	0,6138	0,8509	ns
ACE2-Ab	PCHC	PCS/ME/CFS	0,1295	0,8970	0,9419	ns
ACE2-Ab	PCS/non-ME/CFS	PCS/ME/CFS	0,6341	0,5260	0,7793	ns
ADRA1A-Ab	HC	PCHC	-0,1189	0,9053	0,9419	ns
ADRA1A-Ab	HC	PCS/non-ME/CFS	-0,8932	0,3717	0,6863	ns
ADRA1A-Ab	HC	PCS/ME/CFS	-0,1913	0,8483	0,9387	ns
ADRA1A-Ab	PCHC	PCS/non-ME/CFS	-0,7844	0,4328	0,7364	ns
ADRA1A-Ab	PCHC	PCS/ME/CFS	-0,0733	0,9416	0,9569	ns
ADRA1A-Ab	PCS/non-ME/CFS	PCS/ME/CFS	0,7111	0,4770	0,7574	ns
ADRA2A-Ab	HC	PCHC	-0,1868	0,8518	0,9387	ns
ADRA2A-Ab	HC	PCS/non-ME/CFS	-3,6203	0,0003	0,0035	**
ADRA2A-Ab	HC	PCS/ME/CFS	-3,1379	0,0017	0,0150	**
ADRA2A-Ab	PCHC	PCS/non-ME/CFS	-3,4784	0,0005	0,0055	**
ADRA2A-Ab	PCHC	PCS/ME/CFS	-2,9897	0,0028	0,0197	**
ADRA2A-Ab	PCS/non-ME/CFS	PCS/ME/CFS	0,4887	0,6250	0,8509	ns
ADRB1-Ab	HC	PCHC	-0,9120	0,3618	0,6783	ns
ADRB1-Ab	HC	PCS/non-ME/CFS	-0,8010	0,4231	0,7364	ns
ADRB1-Ab	HC	PCS/ME/CFS	-2,1180	0,0342	0,1206	ns
ADRB1-Ab	PCHC	PCS/non-ME/CFS	0,1124	0,9105	0,9419	ns
ADRB1-Ab	PCHC	PCS/ME/CFS	-1,2218	0,2218	0,5022	ns
ADRB1-Ab	PCS/non-ME/CFS	PCS/ME/CFS	-1,3342	0,1821	0,4391	ns
ADRB2-Ab	HC	PCHC	0,0540	0,9570	0,9570	ns
ADRB2-Ab	HC	PCS/non-ME/CFS	-2,8549	0,0043	0,0258	*
ADRB2-Ab	HC	PCS/ME/CFS	-2,4642	0,0137	0,0610	*
ADRB2-Ab	PCHC	PCS/non-ME/CFS	-2,9469	0,0032	0,0203	*
ADRB2-Ab	PCHC	PCS/ME/CFS	-2,5511	0,0107	0,0516	*
ADRB2-Ab	PCS/non-ME/CFS	PCS/ME/CFS	0,3959	0,6922	0,8744	ns
AGTR1-Ab	HC	PCHC	-0,0710	0,9434	0,9569	ns
AGTR1-Ab	HC	PCS/non-ME/CFS	-1,1974	0,2312	0,5137	ns
AGTR1-Ab	HC	PCS/ME/CFS	-0,7174	0,4731	0,7574	ns
AGTR1-Ab	PCHC	PCS/non-ME/CFS	-1,1411	0,2538	0,5439	ns
AGTR1-Ab	PCHC	PCS/ME/CFS	-0,6549	0,5126	0,7759	ns
AGTR1-Ab	PCS/non-ME/CFS	PCS/ME/CFS	0,4863	0,6268	0,8509	ns
AGTR2-Ab	HC	PCHC	-0,4316	0,6660	0,8580	ns
AGTR2-Ab	HC	PCS/non-ME/CFS	-1,7124	0,0868	0,2315	ns
AGTR2-Ab	HC	PCS/ME/CFS	-0,1325	0,8946	0,9419	ns
AGTR2-Ab	PCHC	PCS/non-ME/CFS	-1,2975	0,1945	0,4575	ns
AGTR2-Ab	PCHC	PCS/ME/CFS	0,3030	0,7619	0,9153	ns
AGTR2-Ab	PCS/non-ME/CFS	PCS/ME/CFS	1,6005	0,1095	0,2856	ns
BDKRB1-Ab	HC	PCHC	-1,8060	0,0709	0,1979	ns
BDKRB1-Ab	HC	PCS/non-ME/CFS	-3,3062	0,0009	0,0095	**
BDKRB1-Ab	HC	PCS/ME/CFS	-3,9840	0,0001	0,0014	***
BDKRB1-Ab	PCHC	PCS/non-ME/CFS	-1,5199	0,1285	0,3282	ns

BDKRB1-Ab	PCHC	PCS/ME/CFS	-2,2065	0,0273	0,1026	ns
BDKRB1-Ab	PCS/non-ME/CFS	PCS/ME/CFS	-0,6866	0,4923	0,7574	ns
CHRM1-Ab	HC	PCHC	-0,6066	0,5441	0,7867	ns
CHRM1-Ab	HC	PCS/non-ME/CFS	-2,4903	0,0128	0,0589	ns
CHRM1-Ab	HC	PCS/ME/CFS	-0,4232	0,6721	0,8580	ns
CHRM1-Ab	PCHC	PCS/non-ME/CFS	-1,9084	0,0563	0,1668	ns
CHRM1-Ab	PCHC	PCS/ME/CFS	0,1857	0,8527	0,9387	ns
CHRM1-Ab	PCS/non-ME/CFS	PCS/ME/CFS	2,0941	0,0363	0,1243	ns
CHRM2-Ab	HC	PCHC	0,4725	0,6366	0,8509	ns
CHRM2-Ab	HC	PCS/non-ME/CFS	-0,5767	0,5641	0,8059	ns
CHRM2-Ab	HC	PCS/ME/CFS	-0,2969	0,7665	0,9153	ns
CHRM2-Ab	PCHC	PCS/non-ME/CFS	-1,0629	0,2878	0,5695	ns
CHRM2-Ab	PCHC	PCS/ME/CFS	-0,7795	0,4357	0,7364	ns
CHRM2-Ab	PCS/non-ME/CFS	PCS/ME/CFS	0,2835	0,7768	0,9153	ns
CHRM3-Ab	HC	PCHC	-0,1499	0,8808	0,9419	ns
CHRM3-Ab	HC	PCS/non-ME/CFS	-1,0593	0,2895	0,5695	ns
CHRM3-Ab	HC	PCS/ME/CFS	-0,3453	0,7299	0,8964	ns
CHRM3-Ab	PCHC	PCS/non-ME/CFS	-0,9212	0,3569	0,6783	ns
CHRM3-Ab	PCHC	PCS/ME/CFS	-0,1979	0,8431	0,9387	ns
CHRM3-Ab	PCS/non-ME/CFS	PCS/ME/CFS	0,7233	0,4695	0,7574	ns
CHRM4-Ab	HC	PCHC	0,0641	0,9489	0,9569	ns
CHRM4-Ab	HC	PCS/non-ME/CFS	-0,3749	0,7078	0,8847	ns
CHRM4-Ab	HC	PCS/ME/CFS	-1,1033	0,2699	0,5682	ns
CHRM4-Ab	PCHC	PCS/non-ME/CFS	-0,4447	0,6565	0,8563	ns
CHRM4-Ab	PCHC	PCS/ME/CFS	-1,1827	0,2369	0,5170	ns
CHRM4-Ab	PCS/non-ME/CFS	PCS/ME/CFS	-0,7379	0,4605	0,7574	ns
CHRM5-Ab	HC	PCHC	-1,9710	0,0487	0,1539	ns
CHRM5-Ab	HC	PCS/non-ME/CFS	-2,4124	0,0158	0,0670	*
CHRM5-Ab	HC	PCS/ME/CFS	-4,9836	0,0000	0,0001	****
CHRM5-Ab	PCHC	PCS/non-ME/CFS	-0,4472	0,6548	0,8563	ns
CHRM5-Ab	PCHC	PCS/ME/CFS	-3,0520	0,0023	0,0171	*
CHRM5-Ab	PCS/non-ME/CFS	PCS/ME/CFS	-2,6048	0,0092	0,0480	*
CHRNA1-Ab	HC	PCHC	-0,1644	0,8694	0,9419	ns
CHRNA1-Ab	HC	PCS/non-ME/CFS	-0,8036	0,4216	0,7364	ns
CHRNA1-Ab	HC	PCS/ME/CFS	-2,6825	0,0073	0,0399	*
CHRNA1-Ab	PCHC	PCS/non-ME/CFS	-0,6475	0,5173	0,7759	ns
CHRNA1-Ab	PCHC	PCS/ME/CFS	-2,5511	0,0107	0,0516	ns
CHRNA1-Ab	PCS/non-ME/CFS	PCS/ME/CFS	-1,9035	0,0570	0,1668	ns
CXCR3-Ab	HC	PCHC	0,6080	0,5432	0,7867	ns
CXCR3-Ab	HC	PCS/non-ME/CFS	-3,7192	0,0002	0,0030	***
CXCR3-Ab	HC	PCS/ME/CFS	-2,4046	0,0162	0,0670	*
CXCR3-Ab	PCHC	PCS/non-ME/CFS	-4,3837	0,0000	0,0006	****
CXCR3-Ab	PCHC	PCS/ME/CFS	-3,0520	0,0023	0,0171	**
CXCR3-Ab	PCS/non-ME/CFS	PCS/ME/CFS	1,3317	0,1830	0,4391	ns
EDNRA-Ab	HC	PCHC	-2,2799	0,0226	0,0875	ns
EDNRA-Ab	HC	PCS/non-ME/CFS	-2,0362	0,0417	0,1353	ns
EDNRA-Ab	HC	PCS/ME/CFS	-2,9649	0,0030	0,0202	*
EDNRA-Ab	PCHC	PCS/non-ME/CFS	0,2468	0,8051	0,9379	ns
EDNRA-Ab	PCHC	PCS/ME/CFS	-0,6940	0,4877	0,7574	ns
EDNRA-Ab	PCS/non-ME/CFS	PCS/ME/CFS	-0,9408	0,3468	0,6713	ns

EDNRB-Ab	HC	PCHC	0,7842	0,4329	0,7364	ns
EDNRB-Ab	HC	PCS/non-ME/CFS	-0,4821	0,6297	0,8509	ns
EDNRB-Ab	HC	PCS/ME/CFS	-0,2820	0,7780	0,9153	ns
EDNRB-Ab	PCHC	PCS/non-ME/CFS	-1,2829	0,1995	0,4605	ns
EDNRB-Ab	PCHC	PCS/ME/CFS	-1,0800	0,2801	0,5695	ns
EDNRB-Ab	PCS/non-ME/CFS	PCS/ME/CFS	0,2028	0,8393	0,9387	ns
F2R/PAR-1-Ab	HC	PCHC	0,1879	0,8510	0,9387	ns
F2R/PAR-1-Ab	HC	PCS/non-ME/CFS	-4,0162	0,0001	0,0014	***
F2R/PAR-1-Ab	HC	PCS/ME/CFS	-1,7176	0,0859	0,2315	ns
F2R/PAR-1-Ab	PCHC	PCS/non-ME/CFS	-4,2591	0,0000	0,0006	***
F2R/PAR-1-Ab	PCHC	PCS/ME/CFS	-1,9304	0,0536	0,1648	ns
F2R/PAR-1-Ab	PCS/non-ME/CFS	PCS/ME/CFS	2,3287	0,0199	0,0795	ns
MAS1-Ab	HC	PCHC	-3,1292	0,0018	0,0150	**
MAS1-Ab	HC	PCS/non-ME/CFS	-3,9227	0,0001	0,0015	***
MAS1-Ab	HC	PCS/ME/CFS	-2,0643	0,0390	0,1300	ns
MAS1-Ab	PCHC	PCS/non-ME/CFS	-0,8039	0,4214	0,7364	ns
MAS1-Ab	PCHC	PCS/ME/CFS	1,0788	0,2807	0,5695	ns
MAS1-Ab	PCS/non-ME/CFS	PCS/ME/CFS	1,8827	0,0597	0,1707	ns
STAB1-Ab	HC	PCHC	-0,6941	0,4876	0,7574	ns
STAB1-Ab	HC	PCS/non-ME/CFS	-2,8022	0,0051	0,0290	*
STAB1-Ab	HC	PCS/ME/CFS	-4,2760	0,0000	0,0006	***
STAB1-Ab	PCHC	PCS/non-ME/CFS	-2,1357	0,0327	0,1189	ns
STAB1-Ab	PCHC	PCS/ME/CFS	-3,6287	0,0003	0,0035	**
STAB1-Ab	PCS/non-ME/CFS	PCS/ME/CFS	-1,4930	0,1354	0,3386	ns

Supplementary table 2: Regression coefficients and p-values for confounder.

AAB target	Group 2	coefficient	min	max	pval	sig
AGTR1	Age	-0,00373	-0,01045	0,002997	0,279041	ns
	Gender	0,202059	0,041029	0,36309	0,015056	up
	Time_since_infection	-0,02665	-0,06952	0,01622	0,224977	ns
AGTR2	Age	0,002971	-0,00625	0,012189	0,528452	ns
	Gender	0,195304	-0,02542	0,416031	0,084935	ns
	Time_since_infection	-0,06724	-0,12601	-0,00848	0,026386	down
EDNRA	Age	-0,00253	-0,00739	0,002323	0,308181	ns
	Gender	0,154432	0,038127	0,270738	0,010184	up
	Time_since_infection	-0,01649	-0,04746	0,01447	0,298137	ns
EDNRB	Age	0,003025	-0,00416	0,010209	0,410462	ns
	Gender	0,135713	-0,03632	0,307747	0,124175	ns
	Time_since_infection	-0,01515	-0,06096	0,030647	0,517643	ns
MAS1	Age	0,003264	-0,00439	0,010913	0,4043	ns
	Gender	0,136215	-0,04695	0,319381	0,147048	ns
	Time_since_infection	-0,04899	-0,09775	-0,00022	0,050816	down
ACE2	Age	0,006741	-0,00446	0,017939	0,239907	ns
	Gender	0,2227	-0,04545	0,490847	0,105671	ns
	Time_since_infection	0,008091	-0,0633	0,079481	0,824501	ns
BDKRB1	Age	0,002579	-0,00503	0,010186	0,507495	ns
	Gender	-0,01883	-0,201	0,16334	0,839716	ns
	Time_since_infection	-0,00589	-0,05439	0,042606	0,812075	ns
F2R	Age	0,004421	-0,00409	0,012931	0,310234	ns
	Gender	0,319658	0,115872	0,523443	0,002507	up
	Time_since_infection	-0,03802	-0,09228	0,016234	0,171642	ns
STAB1	Age	0,007626	-0,00152	0,016774	0,104362	ns
	Gender	0,150528	-0,06852	0,369581	0,180061	ns
	Time_since_infection	-0,04126	-0,09958	0,017055	0,167565	ns
CXCR3	Age	0,002836	-0,0056	0,011275	0,511202	ns
	Gender	0,146535	-0,05557	0,348637	0,15737	ns
	Time_since_infection	-0,04252	-0,09633	0,011287	0,123531	ns
ADRA1A	Age	-0,00282	-0,00982	0,00418	0,43099	ns
	Gender	0,337231	0,169617	0,504844	0,000123	up
	Time_since_infection	-0,03244	-0,07706	0,012187	0,156328	ns
ADRA2A	Age	0,000637	-0,00523	0,006506	0,831889	ns
	Gender	0,081862	-0,05869	0,22241	0,255445	ns
	Time_since_infection	-0,03045	-0,06787	0,006972	0,112863	ns
ADRB1	Age	0,000261	-0,009	0,009527	0,956005	ns
	Gender	0,427119	0,205227	0,649011	0,000232	up
	Time_since_infection	-0,06354	-0,12261	-0,00446	0,036686	down
ADRB2	Age	-0,00111	-0,01235	0,010128	0,846736	ns
	Gender	0,316563	0,047449	0,585677	0,022507	up
	Time_since_infection	-0,00371	-0,07536	0,067934	0,919226	ns
CHRM1	Age	0,002668	-0,006	0,011332	0,547047	ns
	Gender	0,216022	0,008544	0,4235	0,043037	up
	Time_since_infection	-0,04666	-0,1019	0,00858	0,099908	ns
CHRM2	Age	0,002312	-0,00471	0,009333	0,519621	ns
	Gender	0,110748	-0,05738	0,278872	0,198659	ns
	Time_since_infection	-0,05382	-0,09858	-0,00906	0,019727	down
CHRM3	Age	-0,00446	-0,01314	0,004215	0,315079	ns
	Gender	0,284467	0,076674	0,492259	0,008112	up
	Time_since_infection	-0,01108	-0,0664	0,044242	0,695214	ns
CHRM4	Age	-0,0027	-0,01047	0,005066	0,496335	ns
	Gender	0,315241	0,129187	0,501295	0,001127	up
	Time_since_infection	-0,04156	-0,09109	0,007973	0,102173	ns
CHRM5	Age	0,001754	-0,00408	0,007591	0,556727	ns
	Gender	0,097387	-0,04239	0,237164	0,174116	ns
	Time_since_infection	-0,03212	-0,06933	0,005092	0,092761	ns
CHRNA1	Age	0,007424	-0,00035	0,015193	0,063048	ns
	Gender	0,278201	0,092146	0,464256	0,003912	up
	Time_since_infection	-0,0217	-0,07123	0,027837	0,391984	ns

Regression of autoantibodies investigated in study cohorts with confounders age, gender and time since infection. Significance determined by GAMLSS.

Supplementary table 3: Regression coefficients and p-values for study groups (condition vs. reference).

AAB target		HC vs. PCS/ME/CFS	PCHC vs. PCS/ME/CFS	PCS/non-ME/CFS vs. PCS/ME/CFS	PCHC vs. HC	PCS/non-ME/CFS vs. HC	PCS/non-ME/CFS vs. PCHC
ACE2	coefficient	-0.0060	0.0084	-0.0977	0.0144	-0.0917	-0.1061
	p-value	0.9847	0.9638	0.5774	0.9592	0.7710	0.5595
ADRA1A	coefficient	-0.2352	-0.1559	-0.0143	0.0793	0.2209	0.1416
	p-value	0.2291	0.1802	0.8965	0.6525	0.2631	0.2137
ADRA2A	coefficient	0.0867	0.2165	-0.0260	0.1298	-0.1127	-0.2424
	p-value	0.5961	0.0273 (*)	0.7776	0.3797	0.4955	0.0118 (*)
ADRB1	coefficient	-0.1422	0.0189	0.2955	0.1611	0.4376	0.2766
	p-value	0.5821	0.9021	0.0431 (*)	0.4896	0.0948	0.0675
ADRB2	coefficient	0.3994	0.3765	-0.0464	-0.0229	-0.4458	-0.4229
	p-value	0.2034	0.0446 (*)	0.7919	0.9353	0.1600	0.0216 (*)
AGTR1	coefficient	-0.1284	-0.0418	0.0489	0.0866	0.1773	0.0907
	p-value	0.4936	0.7078	0.6423	0.6085	0.3495	0.4066
AGTR2	coefficient	-0.4436	-0.1564	-0.1573	0.2872	0.2863	-0.0009
	p-value	0.0857	0.3068	0.2767	0.2162	0.2707	0.9952
BDKRB1	coefficient	0.3740	0.1852	0.0280	-0.1888	-0.3460	-0.1573
	p-value	0.0793	0.1432	0.8143	0.3243	0.1076	0.2041
CHRM1	coefficient	-0.3031	-0.1663	-0.3094	0.1369	-0.0063	-0.1431
	p-value	0.2105	0.2479	0.0237 (*)	0.5300	0.9795	0.3097
CHRM2	coefficient	-0.3890	-0.0648	-0.0941	0.3242	0.2949	-0.0293
	p-value	0.0482 (*)	0.5778	0.3925	0.0677	0.1370	0.7971
CHRM3	coefficient	-0.0600	-0.1336	0.0679	-0.0737	0.1278	0.2015
	p-value	0.8042	0.3534	0.6175	0.7356	0.6008	0.1540
CHRM4	coefficient	-0.1655	-0.0035	0.1831	0.1620	0.3486	0.1867
	p-value	0.4450	0.9781	0.1337	0.4074	0.1123	0.1403
CHRM5	coefficient	0.2239	0.2468	0.2162	0.0229	-0.0077	-0.0306
	p-value	0.1699	0.0116 (*)	0.0190 (*)	0.8760	0.9626	0.7468
CHRNA1	coefficient	0.2159	0.2954	0.2474	0.0795	0.0315	-0.0480
	p-value	0.3195	0.0229 (*)	0.0434 (*)	0.6842	0.8856	0.7036
CXCR3	coefficient	0.0493	0.3717	-0.1697	0.3224	-0.2189	-0.5414
	p-value	0.8341	0.0086 (**)	0.2003	0.1300	0.3574	0.0001 (***)
EDNRA	coefficient	0.0588	-0.0181	0.1744	-0.0769	0.1155	0.1924
	p-value	0.6639	0.8224	0.0230 (*)	0.5290	0.3986	0.0157 (*)
EDNRB	coefficient	-0.0940	0.0541	-0.0460	0.1482	0.0481	-0.1001
	p-value	0.6386	0.6495	0.6827	0.4124	0.8121	0.3913
F2R/PAR-1	coefficient	0.0018	0.2094	-0.2873	0.2075	-0.2891	-0.4966
	p-value	0.9939	0.1391	0.0324 (*)	0.3327	0.2285	0.0004 (***)
MAS1	coefficient	-0.0490	-0.1796	-0.2251	-0.1306	-0.1762	-0.0455
	p-value	0.8183	0.1578	0.0616	0.4971	0.4137	0.7140
STAB1	coefficient	0.3517	0.5060	0.2476	0.1543	-0.1041	-0.2584
	p-value	0.1691	0.0010 (**)	0.0854	0.5025	0.6861	0.0834

Regression of autoantibodies (-Ab) investigated in PCS patients with and without ME/CFS and healthy controls post or without COVID-19 (PCHC or HC) adjusting for age, gender and time post infection. Significance determined by GAMLSS.
